# Are We Facing a Tsunami of Vaccine Hesitancy or Outdated Pandemic Policy in Times of Omicron? Analyzing Changes of COVID-19 Vaccination Trends in Poland

**DOI:** 10.3390/vaccines11061065

**Published:** 2023-06-05

**Authors:** Marcin Piotr Walkowiak, Jan Domaradzki, Dariusz Walkowiak

**Affiliations:** 1 Department of Preventive Medicine, Poznan University of Medical Sciences, 60-781 Poznań, Poland; 2 Department of Social Sciences and Humanities, Poznan University of Medical Sciences, 60-806 Poznań, Poland; 3 Department of Organization and Management in Health Care, Poznan University of Medical Sciences, 60-356 Poznań, Poland

**Keywords:** COVID-19, vaccination, vaccination coverage, trust in vaccine, social capital, public health, vaccine hesitancy

## Abstract

In this study, we analyzed Polish COVID-19 vaccination data until January 2023 from the European Centre for Disease Prevention and Control to understand individual decision making during the milder Omicron wave. Our findings show a general decline in subsequent vaccine uptake. As the number of government-provided doses increased, completion rates among certain low-risk groups dropped to less than 1%. Elderly individuals, especially those aged 70–79, showed greater adherence but also exhibited decreased interest in subsequent boosters. Healthcare workers exhibited a dramatic shift in their attitude, disregarding the recommended schedule. The overwhelming majority opted out of receiving the second boosters, while the remaining individuals adjusted their timing based on infection trends or the availability of updated boosters. Two factors positively influenced vaccination decisions: societal influence and the availability of updated boosters. Lower-risk individuals were more likely to postpone vaccination until updated boosters were available. Our findings highlight that while Polish policy aligns with international guidelines, it fails to garner significant adherence from the Polish population. Previous studies have shown that vaccinating low-risk groups resulted in more sick days due to adverse events following immunization than the days gained by preventing infection. Consequently, we advocate for the official abandonment of this policy, as its practical abandonment has already taken place, and persisting in pretending otherwise only serves to erode public trust. Therefore, we propose a shift toward treating COVID-19-like influenza with vaccination for vulnerable individuals and those who have close contact with them before the season.

## 1. Introduction

Since the first case of severe acute respiratory syndrome coronavirus (SARS-CoV-2) infection was reported in Poland on 4 March 2020 [1], the government has implemented various public health interventions and control measures, such as the use of personal protective equipment, sanitizers, social distancing, extensive testing, isolation, quarantine and lockdown, to protect the health of Polish citizens and prevent the spread of the virus [2]. Meanwhile, since the World Health Organization (WHO) declared the coronavirus disease of 2019 (COVID-19) a pandemic on 11 March 2020, scientists, the biotechnological industry and pharmaceutical companies worldwide have been working tirelessly to produce effective vaccines against the novel coronavirus. Through intense collaboration, Pfizer BioNTech developed the first COVID-19 vaccine, which was conditionally approved by the European Medicines Agency on 21 December 2020, a year after the first reported case of COVID-19. Subsequently, vaccines made by other companies were also approved, including Moderna, Astra Zeneca and Johnson & Johnson [3].

The Polish National Vaccination Programme, deemed as one of the greatest medical and logistical efforts in the country’s history, began on 27 December 2020, with healthcare workers (HCWs) being prioritized initially. The vaccination of the general population commenced with the elderly, who were followed by other age groups. In May 2021, children aged 16 and 17 were included in the vaccination campaign, and starting from June, the eligibility was extended to children aged 12–15 [4]. The government of Poland initiated a vaccination campaign titled “The Final Stretch” (in Polish: “Ostatnia prosta”) [5], aiming to motivate individuals to get vaccinated with the notion that their vaccination would mark the final step in curbing the pandemic. However, shortly thereafter, the government introduced the administration of first booster shots. Concurrently, as early as the Delta wave, certain scholars noted the emergence of a phenomenon known as “booster hesitancy” [6,7].

Even vaccinated medical students and professionals in Poland were experiencing growing uncertainty about whether receiving another dose of the vaccine was a net benefit or harm to their health. Two groups were particularly hesitant: the first were those who received the AstraZeneca vaccine, which had more reported side effects [8]. The second hesitant group included those who had previously been infected with COVID-19, although at that time, they were dismissed by the literature as relying on “misconceptions of natural immunity triggered by prior infection” [6]. A similar trend emerged in Germany, even among a sample group that was considered to have above-average vaccine acceptance. Individuals who had experienced more severe side effects from the vaccine or who had previously been infected were less likely to be interested in receiving subsequent doses, with as many as 20.4% of them seeking medical assistance due to side effects [7].

From as early as mid-2020, when COVID-19 vaccines were still being developed, studies indicated that individuals were more inclined to vaccinate themselves than their children [9]. Even in late 2021, during the Delta wave, there was an ongoing discussion on child vaccination. The benefits highlighted extended beyond the direct impact of vaccination, emphasizing the gains of reduced transmission. Furthermore, the key advantages mentioned included the avoidance of indirect harms, such as quarantine, school closures, and other negative impacts of lockdown measures. This acknowledgement tacitly recognized the need to consider not only the harm caused by the virus itself but also the potential harms associated with pandemic prevention policies [10]. Some arguments indeed shifted in favor of vaccination, as a year later, it became more difficult to argue about the “unknown long-term safety”, and there were findings that could have enhanced the implementation of developed vaccines: for example, extending intervals to increase immune response [11] and reduce the risk of serious adverse side effects [12]. However, Polish official guidelines continue to recommend a 3-week interval between the first and second doses [13]. Putting aside the perspectives of vaccine-hesitant parents, even from a regulatory standpoint, the vaccines were deemed sufficiently tested to be approved for those age groups relatively late. The vaccination of 5–11-year-olds in Poland started in mid-December 2021, a month and a half before the peak of the Omicron wave, while the vaccination of children aged between six months and 4 years began in mid-December 2022. Furthermore, emerging studies indicated that Omicron was significantly more transmissible than influenza, albeit with a 90% lower fatality rate. These findings significantly weakened arguments pertaining to the prevention of its spread and the benefits of avoiding infection [14].

When the initial wave of Omicron was waning in April 2022, about 92% of the adult population in Poland had detectable anti-spike S1 antibodies. The differences in the detection rates between the vaccinated and unvaccinated individuals were statistically insignificant [15]. People were no longer motivated to avoid seeking medical assistance as isolation and quarantine requirements were lifted [16]. Although mask wearing was still mandatory in medical facilities, there was waning enthusiasm for enforcing the rule.

During the subsequent winter season, the government implemented renewed pandemic prevention measures. General practitioners were provided with new combo tests to detect viruses, including COVID-19. A vaccination promotion campaign called “I want to understand” (In Polish: “Chcę zrozumieć”) was launched on 28 September 2022 [17]. The number of vaccine doses provided reached three doses for children aged six months to 11 years and four doses for older individuals [13].

While the Polish government promotes vaccination for all age groups by emphasizing its effectiveness in preventing severe illness and hospitalization [17], the relevance of this argument becomes questionable in low-risk groups. The provision of subsequent boosters just after three months [13] could be perceived as an implicit admission that the additional protection is short-lived. Furthermore, even the decision to continue the vaccination campaign is no longer straightforward, as exemplified by recent changes in Denmark’s program in summer 2021. Denmark has discontinued offering the primary doses of the COVID-19 vaccine to individuals below 18 years old while restricting boosters to people aged 50 and above, as well as those with underlying health conditions or regular contact with vulnerable populations [18].

An individual trying to make a decision about vaccination may find it challenging to comprehend the current debates, policy shifts, and conflicting approaches taken by different countries. To simplify public health policies, two basic mental models for vaccination could be considered. The first model proposes that achieving herd immunity through universal vaccination is crucial for controlling severe and debilitating diseases. Thus, as a public health policy, universal vaccination is implemented for immunonaive individuals, and failure to comply is considered a violation of social and legal norms. Once fully vaccinated during childhood, the matter is assumed to be resolved. However, while the Polish government technically advises individuals to receive booster shots years later, funding for such boosters is primarily allocated to high-risk groups, suggesting a lack of widespread consideration for their necessity [19]. The second model views the circulation of influenza as a normal occurrence in late winter and early spring. Influenza vaccination is socially and legally regarded as optional. The Polish government fully reimburses the cost of influenza vaccination for high-risk groups, such as old-age pensioners, and provides partial reimbursement for moderate-risk groups such as children [20]. Instead of emphasizing the completion of the vaccination course, individuals are categorized as either vaccinated or not based on whether they received an updated vaccine before the season. Children under the age of 9 are assumed to be immunonaive and are administered 2 doses during their first vaccination season [21,22]. Therefore, natural immunity resulting from prior infections is effectively considered equivalent to a single dose of the vaccine for practical purposes. Apart from distinguishing between children likely to be immunonaive and the rest of the population, there is no encouragement for young, healthy adults, who are highly unlikely to be immunonaive, to catch up on missed doses from previous years within a single season.

The aim of this study is to analyze whether there is a genuine issue of COVID-19 vaccine hesitancy or if it is primarily a result of misclassification due to an outdated official policy. We argue that while many studies refer to vaccine hesitancy when explaining decreasing rates of COVID-19 vaccination, it may result from the outdated pandemic policy. This analysis will be conducted using a proxy indicator, which is vaccine uptake. Although these metrics are theoretically slightly different, for practical purposes, this proxy indicator closely aligns during the analysis period. Given the absence of vaccine shortages, it can be inferred that individuals who desired vaccination were able to receive it. Moreover, it was not viable to designate individuals who finished their initial vaccination course as exhibiting complete vaccine refusal [23]. Thus, it should be stressed that the vaccine uptake we refer to here should not be equated to vaccine hesitancy, as there are many social, cultural, religious and political reasons why people choose not to get vaccinated [24,25,26,27,28].

For example, although according to a social survey conducted in October 2022, 70% of adult Poles declared being vaccinated (27% with the basic dose, 28 with one booster and 15% with two boosters), the number of those unvaccinated increased by 6% to 30%. Moreover, only 13% of respondents were eager to receive another booster, while 42% were not. At the same time, while only 39% of respondents declared being afraid of the pandemic, such worries were more common among those vaccinated (55% vs. 23%) [29]. Another research survey showed that only 38% of Poles believed that COVID-19 vaccination should be compulsory for everyone, and 13% believed it should be compulsory only for particular groups of people, i.e., health service workers (99%), teachers (90%), residential care workers (85%) or uniformed services (73%), and 43% wanted it to be completely voluntary [30].

Thus, while it is commonly assumed that the rates of those unvaccinated reflect the level of vaccine hesitancy resulting either from the fear over the side effects or beliefs in conspiracy theories [6,31,32] it seems that it is actually due to the decreasing morbidity and mortality rates. Most Poles are no longer afraid of the pandemic and have no fear of contracting the coronavirus. In fact, a recent survey conducted in February 2023 demonstrated that coronavirus is starting to be treated like other viruses and that many people were more afraid of contracting influenza than coronavirus (36% vs. 32%) [33]. Poland and other former communist countries had lower vaccination rates before the Omicron outbreak, indicating that vaccine hesitancy was a problem [13]. However, the situation has evolved with the emergence of the milder but more infectious Omicron strain. In the case of vaccine hesitancy, we would expect to observe irrational and risky behaviors. Within a vaccine-hesitant society, HCWs, who are more likely to understand the risks and scientific rationale behind policies should stand out as closely adhering to government guidelines in their individual decisions. People’s vaccination decisions should be influenced by social factors such as peer pressure or maintaining consistency in their choice to either remain unvaccinated or fully vaccinated, with attrition rates at each booster not significantly higher than during primary course. On the other hand, in the case of the emergence of an unofficial policy, observed behaviors would no longer align with the official policy but would still remain rational and loosely follow current scientific understanding. Instead of focusing solely on the number of doses required to be classified as fully vaccinated, individuals would consider various factors such as their risk group, the timing of the infection wave, or the availability of updated boosters. While HCWs, due to their higher job risk profile, should exhibit a higher vaccine acceptance compared to the general population, they would no longer be leaders in following the official policy. Instead, they may become leaders in defying the government in their individual decisions.

## 2. Methods

### 2.1. Data Sources

The preliminary 2021 Census results, as of 28 February 2023, provide information on the composition of the Polish population [34]. However, the reports on vaccination are collected by Polish doctors at vaccination stations via IT system as this information, like any other medical procedures, is available on every patient’s Individual Patient Account (in Polish: Indywidualne Konto Pacjenta). Those reports are than sent to the Ministry of Health and are published on a weekly basis [35]. The Polish government collects digitally recorded medical events and publishes aggregated vaccination data on multiple websites using various methodologies [35,36,37]. However, these sources lack detailed information on boosters or indications of HCW status. Nevertheless, the COVID-19 vaccination data, including boosters and HCW status, are reported to the European Centre for Disease Prevention and Control (ECDC) and were obtained from that source [38]. The vaccination rates are obtained from the ECDS, which is the primary source for weekly COVID-19 data on infections and deaths in the European Union (EU) and European Economic Area (EEA) countries. While the ECDC itself collects the data reports submitted by health authorities from Members States to The European Surveillance System (TESSy), in case the data are incomplete in TESSy, the ESDC collects it from national official sources [39]. However, the ECDC data do not provide information on the exact number of HCWs, and it appears that the Polish government lacks a uniform definition for this group. Initially, the definition was broad, encompassing employees of medical universities and pharmaceutical wholesalers [40]. When mandatory vaccination was introduced at the end of 2022, the government adopted a narrower definition, focusing on those working in medical institutions, pharmacy workers, and medical students [41]. It was later clarified that the obligations also extended to medical employees on maternity leave or administrative workers in medical institutions without patient contact, although enforcement was discretionary [42]. In mid-2023, HCWs eligible for the second booster were defined as those for whom previous doses were mandatory, veterinarians, and those classified as elevated risk by a doctor at the vaccination point [43].

An estimate of the initial vaccination rate among HCWs was obtained from secondary sources. Government statements in the media indicated that a threshold of 90% was reached for this group in mid-2021 [44], while a research sample from that time suggested a rate of 91.2% [8]. These values should be considered as lower bound estimates, as significant increases have been observed in all other groups over the following one-and-a-half years.

### 2.2. Data Analysis

In order to compare the potential divergence between Polish provinces, we selected two provinces that, based on prior studies, exhibited the most contrasting reactions [45]. Mazowieckie Voivodeship is located in central Poland and includes Warsaw, the capital city. While its data were reported to the ECDC in accordance with the Polish administrative division, the area of Warsaw and its suburbs are so well-developed that for reporting purposes to Eurostat, they are excluded and reported as a separate NUTS2 region. On the other hand, Podkarpackie Voivodeship is a rural and economically disadvantaged region, which experienced persistent challenges during the vaccination campaign [45]. To compare the evolving attitudes in these provinces, they will be presented on a graph and segmented by age groups (subject to age data availability in the ECDC). Since the provinces differ not only in size but also in their age composition, a relative chance of getting vaccinated will be used for comparison.

To illustrate the variations among provinces, a relative number of vaccinated individuals is presented. To provide a comprehensive overview and minimize the impact of fluctuations, a 10-week period from 2022-W48 to 2023-W05 is analyzed as a cohesive unit. Previous studies on vaccine uptake have identified the presence of “late adopters”—individuals who exhibit vaccine hesitancy and require more time or social proof before deciding to vaccinate, which is in line with the concept of the innovation adoption curve. These studies have shown notable regional disparities among the late adopters within the youngest and oldest age groups in Poland, while the vaccination pattern among the middle age group appears to be a hybrid of the two [45]. Therefore, for comparative analysis, the populations below 18 years old and those aged 60 and above have been selected.

Groups used for presenting the rates of primary vaccination and booster doses were selected based on data availability in the ECDC, which allowed for an analysis of age groups and HCWs. An individual is considered vaccinated, for the purpose of this study, after receiving the first dose of any COVID-19 vaccine, regardless of the specific vaccination scheme. Treating it otherwise would introduce differentiation based on factors such as receiving a single dose of the Johnson & Johnson vaccine, the most common two-dose regimen, or being a young child eligible for the three-dose regimen as the only option available. For each group, we calculated the proportion of individuals vaccinated, the proportion of vaccinated individuals who received a booster dose, and the proportion of those who received double boosters.

To avoid the post hoc fallacy of interpreting all vaccination after the introduction of the updated booster as a result of waiting for it, it is necessary to distinguish those who intentionally waited from those who were simply unhurriedly planning to vaccinate regardless. One method to achieve this is by utilizing a methodology derived from innovation adoption models that distinguishes late adopters from those who changed their position based on new information [46]. Considering existing factors, such as people gradually overcoming vaccine hesitancy, responding to peer pressure, or finding time to book appointments, the number of newly vaccinated individuals should decrease each subsequent week. This declining phase of the idealized bell curve, occurring after the inflection point at one standard deviation beyond the mean, can be approximated by a summation of shifted negative power functions. In epidemiology, techniques such as mixed linear models are employed to fit functions to phenomena where observations diverge unidirectionally from the general trend, such as during the pandemic wave. Similarly, this technique can be adapted to estimate the number of excess vaccinations that deviate from the innovation adoption model. The waning interest of later vaccine adopters can be approximated using a negative power function. To fit this function to a linear regression model, logarithms of both sides of the equation are taken:

ln(y)=bln(x)+ln(a) 

Directly applying a negative power function assumes an asymptote for *x* = 0, which is unlikely for social phenomena. Therefore, the model is calculated concurrently for various possibilities, shifted by powers of 2, ranging from 1 to 9. All these functions are superimposed and treated as potential explanatory variables in a new model. As this is likely to cause overfitting, the algorithm identifies negative coefficients (*d*) as a clear sign of overfitting. The coefficient with the lowest negative value is eliminated, and the model is refitted. This procedure is repeated until all remaining coefficients (*d*) are positive.

$$y=d_{1}\left(a_{1}{\left(x+2^{1}\right)}^{b_{1}}\right)+d_{2}\left(a_{2}ln{\left(x+2^{2}\right)}^{b_{2}}\right)+\ldots +d_{9}\left(a_{9}ln{\left(x+2^{9}\right)}^{b_{9}}\right)+c$$

This algorithm will be applied for all provinces to all groups that started their vaccination on a mass scale before the introduction of the updated booster, such as HCWs or those aged at least 60, which will be presented graphically. It would allow to assess consistency across provinces, considering their heterogeneity and potential model limitations. The estimates were evaluated in two ways. Firstly, by calculating the standard deviation and confidence intervals for each group, the dispersion of results was examined to assess the generalizability. Secondly, a chi-squared test was conducted to investigate whether age was a statistically significant predictive factor in the decision to wait for the updated vaccine. This test specifically focused on the 60–69 and 70–79 age groups, which began their vaccination simultaneously and could be compared directly.

In order to analyze the response of HCWs, a graphical representation of the number of vaccinations in this group will be presented for the period from 2022-W16 to 2023-W05. The data will be divided into weekly numbers for the first dose, the first booster, and the second booster. These numbers will be scaled so that the week with the highest number of doses in each category is equivalent to 100%. There are three potential mechanisms that could be tested. The enforcement of compulsory vaccination would impact the first dose and the first booster, as only these were compulsory. The increasing infection rate should impact all groups. The availability of updated vaccines should only affect boosters, as they were not used in the primary course.

In cases where differences in selected subsets of the data may not be immediately apparent from a graph, a two-sample Poisson rate test will be employed to determine if these differences are statistically significant and cannot be attributed to chance. The vaccination trend line was calculated using a Python script with the following libraries: pandas 1.4.3, numpy 1.23.2, and statsmodel 0.13.2. The maps were generated using GeoDa 1.20.0.20. A confidence interval of 95% and a significance level of *p* < 0.05 were utilized.

## 3. Results

**Figure 1:**
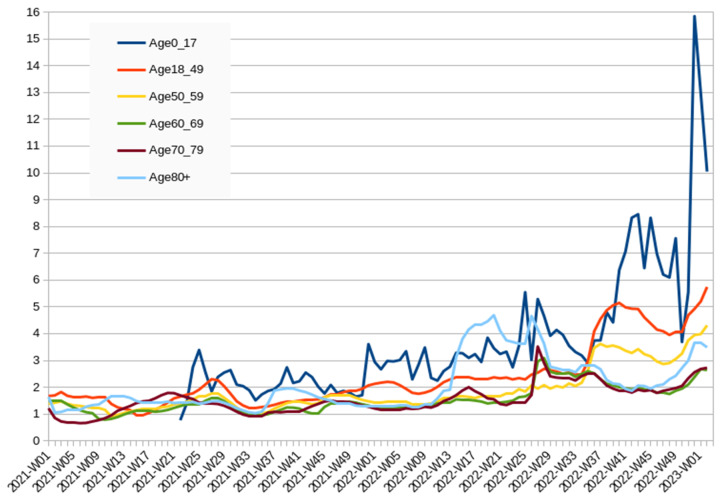
Comparison of COVID-19 vaccination rates between Mazowieckie Voivodeship and Podkarpackie Voivodeship by age group, 2021-W01 to 2023-W05 (smoothed by 5-week average).

Figure 1 compares the weekly ratio of people receiving any dose of COVID-19 vaccine between Mazowieckie Voivodeship and Podkarpackie Voivodeship. The ratio of 1 indicates that both provinces were equally likely to receive a single dose of vaccine in the analysed age groups. During the initial weeks of the vaccination campaign, when vaccine shortages were the main issue, the results were generally comparable, although some subtle differences were noticeable. In the 70–79 age group, Podkarpackie initially had a relatively higher vaccination rate, but in the following months, there was a significant trend reversal. It appears that the higher vaccination rate in this group in the most vaccine-hesitant province was due to the government dividing vaccines equally among regions. The graph indirectly indicates when vaccine shortages ended: as vaccines began to be distributed among younger groups, particularly children.

The steady divergence observed was often punctuated by sudden shifts, which were typically attributed to the introduction of a new age group or dose. As a result, the growing disparities were primarily between new adopters rather than a comparison of late adopters in both provinces. The lower the risk group, the greater the observed divergence, with one notable exception: the group aged 80 and above behaved as if it was lower risk compared to those aged 60–69 or 70–79. While the vaccination data indicate a steady widening of initial differences in children’s vaccination, it is important to acknowledge that some of these differences may have been latent. The introduction of vaccines to successively younger age groups may have simply brought attention to these pre-existing differences.

**Figure 2:**
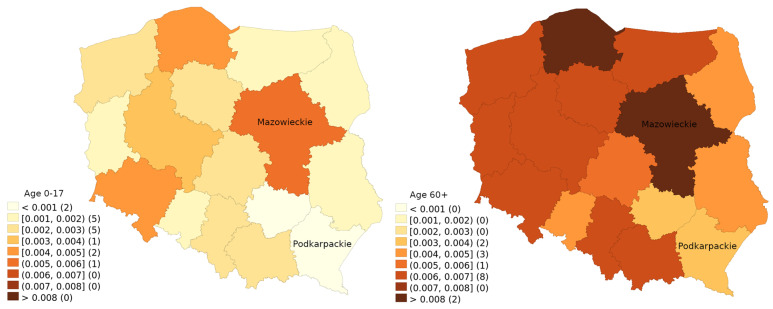
Proportion of the population in age brackets 0–17 and 60 and above who received a dose of the COVID-19 vaccine between 2022-W48 and 2023-W05.

Figure 2 presents the percentage of the population that received a COVID-19 vaccine dose during the ten-week period at the turn of 2022/2023. Notably, the difference in vaccine uptake during this period reached such levels that even children in Mazowieckie Voivodship were more likely to receive a dose than elders in Podkarpackie Voivodship. Given the overall declining interest in vaccination, which increased the impact of random noise, statistical analysis using a two-sample Poisson rate test confirms that this difference in vaccine uptake was statistically significant (*p* < 0.001).

**Figure 3:**
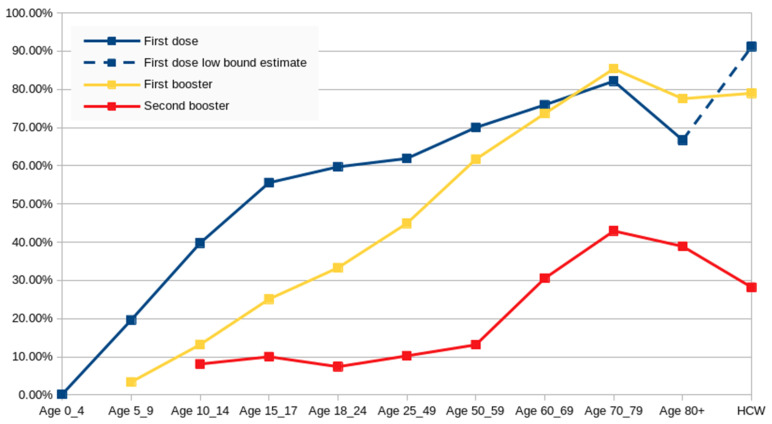
Distribution of the population by vaccination status, including the percentage of individuals who received at least one vaccine dose, the percentage of those who received a single dose and subsequently received a booster, and the percentage of individuals who received a booster and further received a second booster. The data are categorized by age groups and HCWs up to 2023-W05.

Figure 3 displays the overall vaccination rate and the proportion of individuals who received subsequent boosters among those who had received previous doses. These findings reveal a noteworthy contrast with the initial vaccination behavior, as approximately 99% of those who received the first dose of the vaccine completed the full course. However, a significant attrition rate indicates that individuals were not consistently adhering to their initial vaccine acceptance but instead making new decisions with each subsequent booster. Additionally, except for the first booster in groups above 70, each subsequent decision demonstrated an even lower likelihood of opting for the next booster.

There is a notable pattern indicating that the older the person, the less hesitant they were, with the best vaccination rates observed in the 70–79 age group. However, significant challenges were encountered in vaccinating individuals aged 80 and above, and these difficulties worsened with each subsequent dose. HCWs initially demonstrated the highest vaccination rates, with estimates reaching around 90% in summer 2020, even before vaccination and the first booster became mandatory for a portion of this group. Their willingness to receive the first booster declined at a faster rate compared to the 70–79 age group, and their interest in the second booster decreased more rapidly than any group aged 60 and above.

Late vaccination in younger groups yielded minimal results. In the 0.5–4 age group, only 0.16% received the first dose. Among the 10–14 age group, 39.8% received the first dose, of which 13.2% received the first booster, and within that group, 8.1% received the second booster. This cumulative effect led to the second booster being administered to 0.42% of this age group. The number of children up to age 14 who completed all doses for their age group was worryingly comparable to the number of children who annually receive the mandatory chickenpox vaccine [47], even though, according to the law, the chickenpox vaccine is only mandatory for children up to the age of 12 who are immunocompromised or live with immunocompromised individuals [48].

**Figure 4:**
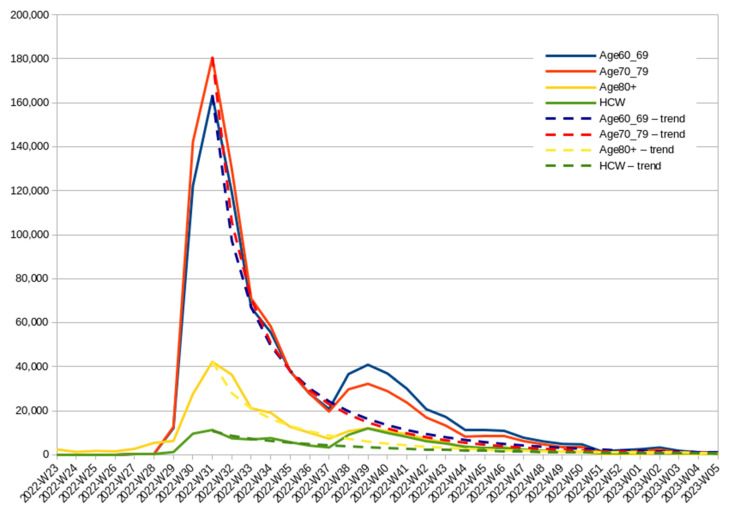
Number of individuals aged 60 and above and HCWs who received the second booster between 2022-W23 and 2023-W05, with a trend line indicating the deviation from the previous trend following the introduction of the bivalent booster.

Figure 4 illustrates a surge in vaccination rates above the trend line following the introduction of a bivalent booster. In comparison to those who were already vaccinated, this booster prompted an additional 8.28% (C.I. 3.87–14.70%) of individuals aged 80 and above, 12.64% (C.I. 9.61–17.15%) of those aged 70–79, 18.23% (C.I. 9.47–21.20%) in the 60–79 age group, and 48.06% (C.I. 28.30–66.25%) of HCWs to receive the updated booster. The wide confidence intervals are a result of the aforementioned divergence between provinces. Notably, individuals aged 80 and above became eligible for the second booster as early as 2022-W16, which may have influenced their relatively lower interest in waiting for the updated booster. Despite this, the increase observed in week W-38 remained statistically significant according to the two-sample Poisson rate test with a *p*-value of less than 0.001. Furthermore, when comparing the reaction of the 60–69 and 70–79 age groups, the application of the Chi-squared test revealed that the percentage of individuals convinced by the updated vaccine was higher in the younger group in a statistically significant manner, with a *p*-value of 0.0124.

**Figure 5:**
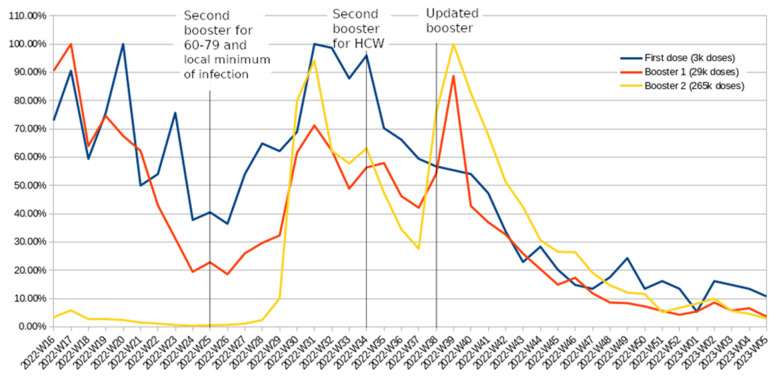
Percentage of HCWs who received their first dose, first booster, or second booster of the COVID-19 vaccine between the period of 2022-W16 and 2023-W05, with all values scaled to the highest number recorded in a single week set as 100%.

Figure 5 displays the relative number of HCWs who received doses of the COVID-19 vaccine starting from late spring 2022. The use of relative terms is employed to highlight the changes over time, considering the significant variation in magnitude. In the initial weeks of the analyzed period, there was a decline in both the number of newly vaccinated HCWs and the reported infection cases. From 2022-W16 onwards, individuals aged 80 and above, along with those with compromised immunity, became eligible for the second booster, resulting in a small peak in second booster administration among HCWs around W18. The announcement of the second booster for age groups 60–79 took place in 2022-W25, coinciding with the period of the lowest number of recorded infections. As infections began to rise in the following weeks, there was a corresponding increase in vaccination enthusiasm among medical personnel, including those who were not yet eligible for vaccination according to policy, although they could have been vaccinated on a case-by-case basis. There was a slight increase in vaccinations during week 2022-W34, after the government officially issued vaccine referrals for the second booster among HCWs. However, a similar increase was also observed for the first booster or even the first dose in that group, which was unaffected by this decision. A more plausible explanation is that the minor dip observed during 2022-W33 was due to a national holiday, as similar vaccination declines were observed during Christmas and the New Year [49]. Based on these data, it can be concluded that the policy change had no discernible impact on vaccination rates in contrast to the impact of the infection wave or the introduction of an updated vaccine.

While the graph indicates the introduction of the bivalent booster in 2022-W38, the accessibility varied. An insignificant amount of the new booster was already delivered toward the end of the previous week. During 2022-W38, both types of boosters were being administered, and the Ministry of Health spokesman publicly criticized patients for attempting to select the new booster, claiming that both types were equally effective [50]. The results of a two-sample Poisson rate test indicate a statistically significant increase (*p* < 0.001) in booster uptake among HCWs in 2022-W38. Importantly, it is unlikely that any other factors were driving this increase in vaccination, as there was actually a decline in the uptake of the primary vaccination course that was not updated.

## 4. Discussion

This study highlights a growing discrepancy in Poland between the official COVID-19 policy and individual attitudes toward vaccination [29,30,33]. The observed lack of interest among low-risk groups, combined with the increasing divergence in policies, does not justify the allocation of further resources. Instead, public authorities should prioritize their attention on high-risk groups. There is a worrisome decline in interest in subsequent boosters among individuals aged 80 and above. Additionally, it appears that younger high-risk groups are also being overlooked. While we are unable to directly identify them from aggregated data, we infer their existence from the fact that the number of pre-adolescent children who have completed all doses of the COVID-19 vaccine is comparable to the annual number of pre-adolescent children who receive the compulsory chickenpox vaccine due to their classification as being at high immunological risk [47,48].

In the initial phase of the pandemic, there was an observed tendency for countries to “group think” in introducing lockdowns in reaction to their neighbors doing so [51], even though just before the COVID-19 pandemic, lockdowns were being discouraged by the WHO as a pandemic prevention policy because they were considered too costly and disruptive in relation to potential gains [52]. Similar social dynamics become visible in the case of vaccination. As the most recent WHO Vaccination Strategy states: “COVID-19 vaccine and immunization progress needs to be sustained and momentum enhanced” [53]. Nevertheless, advice issued in April 2023 by international bodies concerning the number of doses, if any, that low-risk groups should receive is becoming increasingly elusive. The WHO advises to: “Take all COVID-19 vaccine doses recommended to you by your health authority” [54], while the ECDC recommends the vaccination of “target groups suited for the local epidemiological context” [55]. This level of blanket approval for mutually contradictory policies concerning low-risk groups can be interpreted in two ways. It could suggest that despite over a year having passed since the Omicron outbreak, the available research remains genuinely inconclusive. Alternatively, it raises the possibility that factors beyond scientific considerations, such as political or institutional dynamics, may have also influenced the decision-making process.

According to the Polish state policy, the COVID-19 pandemic is still considered ongoing, and there is even formally an escalation in the vaccination efforts compared to the times of the more severe Delta variant. This is evident through the continuous expansion of age groups and the increasing number of recommended doses for low-risk groups. On the other hand, individuals in working age exhibited a lack of interest in pursuing further vaccination for themselves or their children. This trend cannot be easily attributed to vaccine hesitancy among the misinformed general public, as there was also a considerable decrease observed among HCWs. Through their individual decisions, they have essentially revised the healthcare policy, adopting a strategy for COVID-19 vaccination reminiscent of influenza vaccination, whereby an updated vaccine is pursued prior to the start of the season.

According to the ECDC guidelines, which adopt a broad definition of vaccine hesitancy from the WHO, the recommended approach is to provide vaccine-hesitant individuals with reliable information and educate them about the favorable risk–benefit ratio [56]. This advice presupposes that being classified as vaccine-hesitant necessitates doubting the vaccine, even when the scientific data overwhelmingly support its favorable risk–benefit ratio. However, even before the emergence of the Omicron variant, the vaccination of adolescents was already a topic of controversy. Some studies found a favorable risk–benefit ratio for immunonaive girls, comparing the rare risk of myocarditis from vaccination to the rare risk of hospitalization from COVID-19 [57], while others found a favorable ratio for the entire adolescent population [58]. For low-risk individuals, the argument that the benefits outweigh the risks of Omicron is not supported by available studies, as the overall likelihood of experiencing a systemic reaction after receiving a booster is approximately 15.9% with diminishing effectiveness against infection after 5–8 months [59]. A retrospective study on the Swedish population revealed that 767 individuals with natural immunity would need to be fully vaccinated to prevent a single case of COVID-19 [60]. Additionally, vaccinated individuals faced an average of 4.4 symptomatic days if they contracted the Omicron variant [61]. Using these numbers for a rough estimate, considering natural immunity to be at least as effective as vaccine-derived immunity [60] and ac: reference counting for only the side effects of a single dose, each day prevented from infection would come at the cost of approximately 27.6 days of being sick from side effects. These calculations are highly approximate, and they do not account for other potential negative impacts.

Regardless of whether the low-risk group could benefit from additional vaccination in general, it is even more challenging to argue that they would benefit from the exact same frequency of boosters as elderly individuals, as the current policy implicitly assumes. Beyond the direct benefits associated with risk profiles, in the younger groups, not only does their immunity tend to last longer [59], but they also experience a higher incidence of adverse side effects compared to older age groups [58]. In certain cases, there were significant disparities—after the first dose of the Pfizer vaccine, systemic side effects were reported by 20.7% of individuals aged 55 or younger, while only 10.6% of those over 55 reported such effects [58].

While evidence from Polish sewage data was limited [62], it roughly aligned with the pandemic situation, except for a gradual divergence observed between virus concentration in sewage and other metrics, such as infection cases, officially recorded deaths, or overall mortality. This led to a paradoxical situation where, during most of the second half of 2022, virus concentration was higher than during the peak of the Delta wave. It is difficult to ascertain whether the individuals responsible for this high concentration were even symptomatic, as they clearly were not diagnosed. Sewage data would indicate that the initial Omicron infection wave (2022-W02 to 2022-W16), where the unvaccinated population reached a similar likelihood of having antibodies as the vaccinated, only accounted for half of the infections throughout the year. This sustained level of infection suggests a high level of population immunity. While acknowledging the issue of under-detection in COVID-19 cases, including evidence suggesting significant undercounting of deaths [63], other proxy data support the overall trend of a diminishing impact of COVID-19. Notably, apart from the end of the Delta wave in 2022-W01, the second deadliest week occurred in 2022-W52 [64], coinciding with the peak of influenza A infections reported by the ECDC [65].

Viewing COVID-19 in a manner akin to influenza does not imply assuming its benign nature, as influenza itself causes a deadly season annually and has historically led to pandemics with millions of deaths, including those in 1957 and 1968 [66], which are barely remembered today. Persisting with public health policies that are so widely ignored, as if they were perceived as detached from reality, not only fails to achieve their intended objectives but also erodes trust. This is particularly concerning considering the significant hindrance lack of trust in public institutions posed in pandemic response, which was especially pronounced in Eastern Europe [67,68].

A similar pattern was observed in countries that initially fared better than Poland, such as Austria and Italy, where it was termed “vaccine fatigue” [69]. The observed trends in those countries, such as a general loss of interest, low impact of campaigns or incentives, and some moderate interest in receiving an updated booster, aligned closely with our findings. However, their interpretation of these findings favored intensifying campaigns emphasizing the sense of community. While we recognize the significance of social factors in vaccination campaigns, it is likely that these countries are following a trajectory similar to Poland, albeit with a slight lag.

Encouraging HCWs to continue approaching COVID-19 similarly to annual influenza, as they are already doing, should be accepted as an official policy. Given the sudden surge in infections caused by COVID-19 in late summer, it is necessary to implement a more flexible response and vaccination approach. Considering that already a year ago, both the unvaccinated and vaccinated populations had an equal likelihood of having antibodies [15], and natural immunity is proven to be at least as effective as vaccination [60], there is no need to pretend that these two groups differ. Additionally, it is time to acknowledge that low-risk individuals with natural immunity are highly unlikely to benefit from quickly recouping the four missed doses.

While the risk–benefit ratio may not appear favorable among the young population in general, there is an additional consideration to take into account. If they are likely to become a source of infection for vulnerable individuals, the modest chance of systemic side effects becomes less significant. However, despite making special accommodations for HCWs, Polish COVID-19 guidelines do not extend similar considerations to household members of highly vulnerable individuals. Particularly if the mass vaccination of low-risk groups is officially discontinued, it is crucial to regularly remind this specific group that their risk–benefit ratio differs from that of the general population.

## 5. Limitations

Inferring general opinions from the actual behaviors of the entire population is likely to yield more reliable results compared to surveying a limited sample This approach offers the advantage of circumventing social desirability bias, particularly in the aftermath of extensive social campaigns. However, it is important to acknowledge that studying actions provides valuable insights but may not capture the underlying arguments and decision-making processes. Additionally, it does not fully account for individuals’ potential uncertainty or regret regarding their choices.

Given their professional background in healthcare, it is reasonable to expect that HCWs in general possess a fundamental understanding of medical issues related to their own well-being. However, it is important to acknowledge the limitations of rational decision-making processes [70]. Furthermore, in models assuming perfect information and rationality, individuals prioritize self-interest, overlooking externalities such as the impact on the spread of infectious diseases, resulting in socially sub-optimal outcomes.

## 6. Conclusions

Previous studies indicate that individuals, regardless of their vaccination status, are unlikely to be immunonaive, and for low-risk groups, the ratio of days spent being sick from systemic side effects compared to the days of sickness prevented from infection is unlikely to be favorable. Despite this, the official policy expects individuals to catch up on a steadily increasing number of missed vaccine doses. In practice, people already tend to treat COVID-19 similarly to influenza, which involves the annual vaccination of high-risk groups before each season. From a policy perspective, updating the vaccine could be necessary to convince medium-risk individuals, even if the medical gains from the new formula may not be substantial. It is crucial for public policy to acknowledge and reflect this reality, as failing to do so erodes public trust, which was a significant issue during the early days of the pandemic.

## Data Availability

The data are available at: https://vaccinetracker.ecdc.europa.eu/public/extensions/COVID-19/vaccine-tracker.html#uptake-tab, https://bdl.stat.gov.pl/bdl/start (accessed on 12 March 2023).

## Abbreviations

COVID-19	the coronavirus disease 2019
ECDC	European Centre for Disease Prevention and Control
WHO	World Health Organization
